# Supplementary material for: Detection of Salmonella Pathogenicity Islands and Antimicrobial-Resistant Genes in Salmonella enterica Serovars Enteritidis and Typhimurium Isolated from Broiler Chickens
Source: Antibiotics (Basel). 2024 May 16;13(5):458. doi: 10.3390/antibiotics13050458 (PMC11117945; doi:10.3390/antibiotics13050458)
Supplement: Supplementary file 1 [file antibiotics-13-00458-s001.zip › antibiotics-2992427-supplementary.pdf]

Supplementary Materials

Table S1. List of antibiotic resistance genes primers used in this study.

Class	Target gene	Primer	Primer sequence (5′ → 3′)	Amplicon size (bp)	Annealing temp (°C)	References
**Tetracycline**	*tet*(A)	TETA-F<br>TETA-R	GCGCTNTATGCGTTGATGCA<br>ACAGCCCGTCAGGAAATT	387	62	Jiang et al., 2013
	tet(O)	TETO-F<br>TETO-R	ACGGARAGTTTATTGTATACC<br>TGGCGTATCTATAATGTTGAC	171	60	Aminov et al., 2001.
	tet(X)	TETX-F<br>TETX-R	CCGACACGGAAGTTGAAGAA<br>CCTTGGTGAGATGCCATTAGC	468	60	Aminov et al., 2001.
	tet(P)	TETP-F<br>TETP-R	CTTGGATTGCGGAAGAAGAG<br>ATATGCCCATTTAACCACGC	676	60	Hong et al., 2018
	tet(W)	TETW-F<br>TETW-R	GAGAGCCTGCTATATGCCAGC<br>GGGCGTATCCACAATGTTAAC	168	50	Jiang et al., 2013
	tet(K)	*tet*(X)-F<br>*tet*(X)-R	TCGATAGGAACAGCAGTA<br>CAGCAGATCCTACTCCTT	169	61	Hong et al., 2018
**Colistin**	*mcr*-1	mcr-1-F<br>mcr-1-R	TATCGCTATGTGCTAAAGCCTG<br>CGTCTGCAGCCACTGGG	1139	56	Jousset et al., 2019
	mcr-2	mcr-2-F<br>mcr-2-R	TATCGCTATGTGCTAAAGCCTG<br>AAAATACTGCGTGGCAGGTAGC	816	56	Jousset et al., 2019
	mcr-3	mcr-3-F<br>mcr-3-R	CAATCGTTAGTTACACAATGATGAAG<br>AACACATCTAGCAGGCCCTC	676	56	Jousset et al., 2019
	mcr-4	mcr-4-F<br>mcr-4-R	ATCCTGCTGAAGCATTGATG<br>GCGCGCAGTTTCACC	405	56	Jousset et al., 2019
	mcr-5	mcr-5-F<br>mcr-5-R	GGTTGAGCGGCTATGAAC<br>GAATGTTGACGTCACTACGG	207	56	Jousset et al., 2019
**Sulfonamide**						
	*sul*I	sulI-F<br>sulI-R	CGCACCGGAAACATCGCTGCAC<br>TGAAGTTCCGCCGCAAGGCTCG	163	63	Hong et al., 2018
	*sul*II	sulII-F	TCCGGTGGAGGCCGGTATCTGG	191	63	Hong et al., 2018

		sulII-R	CGGGAATGCCATCTGCCTTGAG			
	*sul*III	sulIII-F<br>sulIII-R	TCCGTTCAGCGAATTGGTGCAG<br>TTCGTTCACGCCTTACACCAGC	128	61	Hong et al., 2018
**β-lactam**						
	*amp*C	AmpC -F<br>AmpC R	GTGACCAGATACTGGCCACA<br>TTACTGTAGCGCCTCGAGGA	822	61	Liu et a., 2018
	SHV	SHV-F<br>SHV-R	CACTCAAGGATGTATTGT G<br>TTAGCGTTGCCAGTGCTCG	885	55	Ramatla et al., 2022
	OXA	OXA-F<br>OXA -R	ACACAATACATATCAACTTCGC<br>AGTGTGTTTAGAATGGTGATC	813	55	Ramatla et al., 2022
	CARB	CARB-F<br>CARB-R	CAAGTACTTTYAAAACAATAGC<br>GCTGTAATACTCCKAGCAC	534	46	Jiang et al., 2013
	TEM	TEM-F<br>TEM-R	TTC TTG AAG ACG AAA GGG C<br>ACGCTCAGTGGAACGAAAAC	1150	55	Ramatla et al., 2022
**Aminoglycoside**						
	*str*A	strA-F<br>strA-R	CTTGGTGATAACGGCAATTC<br>CCAATCGCAGATAGAAGGC	548	55	Hong et al., 2018
	*str*B	strB-F<br>strB-R	ATCGTCAAGGGATTGAAACC<br>GGATCGTAGAACATATTGGC	509	56	Hong et al., 2018
	*aad*A	aadA-F<br>aadA-R	ATCCTTCGGCGCGATTTTG<br>GCAGCGCAATGACATTCTTG	283	56	Hong et al., 2018
	aadE	aadE-F<br>aadE-r	ATGGAATTATTCCCACCTGA<br>TCAAAACCCCTATTAAAGCC	386	50	Hong et al., 2018

Supplementary References

1 Sun W, Qian X, Gu J, Wang XJ, Duan ML. Mechanism and effect of temperature on variations in antibiotic resistance genes during anaerobic digestion of dairy manure. Scientific reports. 2016;6(1):30237.

2 Jiang L, Hu X, Xu T, Zhang H, Sheng D, Yin D. Prevalence of antibiotic resistance genes and their relationship with antibiotics in the Huangpu River and the drinking water sources, Shanghai, China. Science of the Total Environment. 2013;458:267-72.

3 Hong B, Ba Y, Niu L, Lou F, Zhang Z, Liu H, Pan Y, Zhao Y. A comprehensive research on antibiotic resistance genes in microbiota of aquatic animals. Frontiers in Microbiology. 2018;9:1617.

4 Ramatla T, Mileng K, Ndou R, Mphuti N, Syakalima M, Lekota KE, Thekisoe OM. Molecular detection of integrons, colistin and β-lactamase resistant genes in Salmonella enterica serovars enteritidis and typhimurium isolated from chickens and rats inhabiting poultry farms. Microorganisms. 2022;10(2):313.

5 Gundran RS, Cardenio PA, Villanueva MA, Sison FB, Benigno CC, Kreausukon K, Pichpol D, Punyapornwithaya V. Prevalence and distribution of bla CTX-M, bla SHV, bla TEM genes in extended-spectrum β-lactamase-producing E. coli isolates from broiler farms in the Philippines. BMC veterinary research. 2019;15:1-8.

6 Liu G, Ding L, Han B, Piepers S, Naqvi SA, Barkema HW, Ali T, De Vliegher S, Xu S, Gao J. Characteristics of Escherichia coli isolated from bovine mastitis exposed to subminimum inhibitory concentrations of cefalotin or ceftazidime. BioMed Research International. 2018;2018.

7 Xu J, Xu Y, Wang H, Guo C, Qiu H, He Y, Zhang Y, Li X, Meng W. Occurrence of antibiotics and antibiotic resistance genes in a sewage treatment plant and its effluent-receiving river. Chemosphere. 2015;119:1379-85.
